# Antimicrobials in Livestock Farming and Resistance: Public Health Implications

**DOI:** 10.3390/antibiotics14060606

**Published:** 2025-06-14

**Authors:** Marilena Trinchera, Silvia De Gaetano, Elenoire Sole, Angelina Midiri, Serena Silvestro, Giuseppe Mancuso, Teresa Catalano, Carmelo Biondo

**Affiliations:** 1 Department of Human Pathology, University of Messina, 98125 Messina, Italy; tmarilena08@gmail.com (M.T.); sdegaetano6@gmail.com (S.D.G.); elenoiresole@icloud.com (E.S.); amidiri@unime.it (A.M.); mancusog@unime.it (G.M.); 2 IRCCS Centro Neurolesi Bonino Pulejo, 98124 Messina, Italy; serena.silvestro@irccsme.it; 3 Department of Clinical and Experimental Medicine, University of Messina, Via Consolare Valeria, 98125 Messina, Italy; teresa.catalano@unime.it

**Keywords:** intensive livestock farming, transmission of multidrug resistance, antimicrobial resistance policies

## Abstract

The accelerated spread of bacterial resistance has been demonstrated to reduce the effectiveness of antibiotic treatments for infections, resulting in higher morbidity and mortality rates, as well as increased costs for livestock producers. It is expected that the majority of future antimicrobial use will be in animal production. The management of antimicrobial resistance (AMR) in the livestock sector poses significant challenges due to the multifaceted nature of the problem. In order to identify appropriate solutions to the rise of antimicrobial resistance, it is imperative that we have a comprehensive understanding of the disease dynamics underpinning the ways in which antimicrobial resistance is transmitted between humans and animals. Furthermore, in consideration of the anticipated requirement to satisfy the global demand for food, it is imperative that we guarantee that resistance is not transmitted or propagated during the treatment and disposal of animal waste, particularly from intensive farming. It is also crucial to formulate a research agenda to investigate how antibiotic resistance in animal faeces from livestock farming is affected by intensified farming activities. The review analyses the environment’s role in the transmission resistance chain and reviews methodologies for disrupting the link. A particular focus is placed on the limitations of the applied methodologies to reduce antimicrobial resistance in global animal production.

## 1. Introduction

### 1.1. Antibiotics and Antibiotic Resistance

During the 20th century, scientists discovered molecules that could fight off infections. This made people hopeful that new treatments would be developed [1]. The utilisation of antibiotics as a pharmaceutical intervention for the management of bacterial infections in humans and animals can be traced back to the early and middle 20th century, respectively [2]. However, Fleming’s predictions were soon corroborated, as within a few decades, microbes had developed resistance to the drugs, thereby rendering the majority of them ineffective for treating diseases [3]. In less than a hundred years, the world has witnessed a global health crisis of unprecedent proportions, with multidrug resistance significantly impacting global socio-economic development [4]. Studies have shown that antibiotic resistance is an inherent feature of microorganisms, with evidence found in samples from glacial waters over two millennia old, and, thus, in environments unaffected by humans [5]. Nevertheless, the emergence of bacterial resistance is a direct consequence of the overuse of antibiotics. It is evident that, notwithstanding the inevitable occurrence of this phenomenon as a result of the process of natural selection—in which bacteria adapt in order to evade the effects of drugs, the rate of propagation of this event is a direct consequence thereof. This practice is particularly prevalent within the sphere of animal husbandry [6,7].

### 1.2. The Silent Pandemic

AMR carries profound implications for global public health, resulting in elevated healthcare costs, prolonged hospital stays, and, most significantly, increased mortality for those infected by a drug-resistant pathogen [8]. In order to comprehend the significance of this phenomenon, it is crucial that we recognise that, in 2019, the number of deaths attributable to this issue exceeded 1.2 million, a figure that is projected to escalate to 10 million by the year 2050 [9,10]. Within the European Union, the mortality rate due to antibiotic resistance in 2020 exceeded 35,000, with Italy accounting for nearly one-third of these casualties [11]. The term ‘silent pandemic’ has, thus, been coined to denote the gravity of this situation [12]. The increased mutation and mobilisation of DNA at low antibiotic concentrations has also been demonstrated to result in antimicrobial resistance, thereby allowing microorganisms to adapt and evolve [3]. These genes evolve in a competitive environment, countering other strains in order to ensure their own survival. Chromosomal DNA has the capacity to convey resistance genes via mutations; however, the latter are predominantly located on mobile elements, such as transposons and plasmids, which can facilitate the spread of resistance genes [13]. Once a pathogen has acquired these genes, eradication becomes challenging, particularly if the gene does not impose a significant metabolic burden [14]. It is important to note that a limit exists for the cost associated with the maintenance of these resistance genes. Therefore, they may not become firmly established within the bacterial population, unless there is a significant environmental selective pressure for their maintenance [15]. It is, therefore, evident that the development and expansion of new resistances is contingent on the environment, where selective pressure encourages gene transfer and maintenance [16].

### 1.3. Global Antimicrobials Use in Livestock Farming

The utilisation of antibiotics in healthcare and agriculture, specifically in intensive livestock farming, is problematic as it can result in the emergence of novel resistance due to the misuse and overuse of these antimicrobials [8]. A number of factors have been identified that, by applying a form of selective pressure, favour the transfer of resistant pathogenic bacteria and/or resistance genes, affecting various sectors across the board [17]. Industrial waste, especially in the pharmaceutical industry, leads to the release of significant quantities of molecules with antibiotic activity into the environment [18]. The inadequate management of wastewater, including that from both agricultural activities and residential areas, plays a pivotal role in ecosystem contamination. This phenomenon can be further exacerbated by the irrigation of agricultural fields, thereby contributing to the dissemination of antibiotic-resistant bacteria [19]. The expansion of antimicrobial resistance is also influenced by globalisation, which has resulted in the international marketing of agricultural products, livestock, and/or their products and derivatives [20]. In addressing the emergence of the multidrug resistance (MDR) threat, it is imperative to recognise the multifactorial nature of the problem, in which the health of humans, animals, and the entire ecosystem are inextricably linked. This review provides a comprehensive overview of the utilisation of antibiotics in livestock farming (excluding aquaculture) and its role in the propagation of antibiotic resistance. The mechanisms driving antimicrobial resistance and potential policy interventions to mitigate this global public health threat are further discussed.

## 2. The Use of Antibiotics in the Veterinary Sector and in the Livestock Industry

The overuse and/or misuse of antibiotics is the primary cause of AMR [21]. This misuse occurs in all three principal areas: human, veterinary, and environmental [21]. The solution to this problem is straightforward: their usage must be reduced and reserved solely for essential applications in all three areas. The veterinary sector has frequently been cited as a significant contributor to the phenomenon of antibiotic resistance [22]. It has been estimated that more than 50% of antibiotics used globally are used in veterinary medicine [23,24]. Antibiotic-resistant bacteria, including *Escherichia coli*, *Salmonella*, *Campylobacter*, and enterococci, have demonstrated the ability to cross the species barrier and infect humans, often through direct contact, via the food chain, or by the transfer of resistance genes (plasmids) from animal bacteria to common human pathogens, particularly those residing in the intestinal tract [25,26,27]. The emergence of a resistance to antibiotics is an inherent property of bacterial organisms. Nevertheless, the temporal interval between the introduction of ampicillin (one of the most prevalent penicillins) into the human therapeutic market and the initial manifestation of resistance by the genus *Salmonella* to this antibiotic was too brief (a mere 12 months) for the phenomenon to be ascribed exclusively to the prescription practices of medical professionals [28]. To this end, a comprehensive investigation was conducted on a multitude of *Salmonella* strains, obtained from over 30 countries and diverse origins (humans, farm animals, feed, and food), using whole-genome sequencing. This investigation revealed that the ampicillin resistance gene (*blaTEM-1*) emerged in humans in the 1950s, prior to the introduction of the antibiotic in the pharmaceutical market [28]. It can, thus, be deduced that the addition of penicillin to animal feed in the decade leading up to its introduction for human use may have contributed to the spread of resistance [3,27]. These findings suggest that antibiotic residues in soil, wastewater, and livestock manure may have a significantly greater impact on the spread of resistance than was previously thought. The utilisation of antibiotics in veterinary medicine is subject to stringent regulation in numerous countries, frequently exceeding the regulatory standards observed in human medicine [29]. However, the utilisation of antibiotics in veterinary medicine initially followed the same trajectory as in human medicine [30]. Examples of antibiotics used in both human medicine and veterinary medicine include tetracyclines and penicillin [23]. In the USA, oxytetracycline is still used, but only for therapeutic use in pig feed, with concentrations ranging from 200 to 800 mg/kg [31]. When employed for growth-promoting purposes, concentrations range from 50 to 200 mg/kg. Table 1 shows the most common types of antibiotics used in veterinary medicine.

Conversely, other antibiotics, not employed in a medicinal context, are specifically utilised in animal feed for their effect on growth (e.g., avilamycin, avoparcin, and flavomycin) [40]. In contrast, Europe and numerous other countries have regulations that prohibit the use of antibiotics registered for therapeutic use in humans and animals as growth promoters [41].

## 3. The Key Reasons for Using Antibiotics in the Field of Animal Husbandry

Antibiotics have long been used to promote growth, but we do not know much about the metabolic pathways they affect. The current leading theory is related to the gut microbiota and how it relates to productivity. Research has shown that low-dose antibiotics, without killing the beneficial bacteria needed for efficient nutrient absorption, can change the microbiota [42]. Studies have also shown that not all antibiotics stimulate growth equally. For example, virginiamycin enhances growth, while tetracycline has a low yield. This suggests that they interact differently with the microbiota. Virginiamycin appears to reduce the microbiota’s nutrient needs by changing the abundance of its component genera [43]. The use of antibiotics in the field of veterinary medicine is principally driven by three factors: firstly, the treatment of specific infectious diseases (i.e., third-generation cephalosporins are employed in the treatment of pneumonia in all mammalian food-producing species [44]); and, secondly, control or metaphylactic use—the treatment of a group of animals after disease has been diagnosed in part of the group. The objective of this intervention is to treat animals that are manifesting clinical signs of illness, with the aim of preventing the spread of disease to animals in close proximity that may already be infected (i.e., chlortetracycline, a tetracycline antibiotic, is used to treat bovine respiratory disease, especially in high-risk calves [45]). The third category, preventative or prophylactic, involves the treatment of an animal or group of animals before clinical signs of disease occur (i.e., tetracyclines are widely used in the livestock industry to prevent diseases [46,47]. Growth-promoting antibiotics are extensively employed in the farming of pigs, chickens, and calves in countries such as Myanmar, Costa Rica, and Canada. A comprehensive understanding of the mode of action of such antibiotics can be obtained from the observation that the growth of germ-free animals is 20% higher than the growth of animals that have been left free and not received growth-promoting antibiotics in their diet [3].

The population has exhibited steady growth, with the economy, particularly in developing countries, demonstrating concomitant expansion, resulting in augmented buying capacity. This has resulted in an increased demand for food, especially animal proteins, compared to previous decades [48]. In response to this mounting demand, there has been a transition towards more expansive agricultural production methods. This transition has been primarily characterised by intensive farming systems, particularly for cattle, poultry, and pigs [49]. The underlying objective of this transition is to reduce costs by rendering this category of products suitable for large-scale consumption. The evolution of animal production systems has resulted in two key objectives: the optimisation of efficiency and the implementation of stringent disease prevention measures. Concurrently, there has been an increase in the utilisation of antibiotics, employed to prevent the occurrence of outbreaks on the farm, thereby averting substantial economic losses [7]. Antimicrobials have played a pivotal role in the prevention, treatment, and control of food-animal diseases caused by pathogens such as *E. coli*, *S. aureus*, *Campylobacter* spp., and *Salmonella* spp. [50]. Nevertheless, the widespread use of veterinary antimicrobials has favoured the diffusion of antimicrobial resistance, with important consequences for animal and, potentially, human health. A research study in the Pothohar region of Pakistan found that a significant number of *Staphylococcus aureus* isolates that cause subclinical bovine mastitis are resistant to multiple drugs and methicillin [51]. Indeed, the utilisation of antimicrobials in food-producing animals has a long history, dating back to 1951 when the Food and Drug Administration authorised their use as additives in animal feed in the USA, without the need for a veterinary prescription [52]. Since then, antimicrobials have been employed for many decades, not only for the treatment of animal infections, but also for other applications. It is estimated that over 70% of global antimicrobials are used in food-producing animals, particularly in nations with substantial livestock industries, including China, Brazil, and the USA [52,53,54].

### 3.1. Therapeutic Use

The therapeutic use of antibiotics in animals refers to the administration of antimicrobials for the treatment of animals with clinically diagnosed infectious diseases [23]. A wide range of antibiotics have been approved for this purpose, including for respiratory diseases, enteric diseases, and mastitis, with many benefits for both animal health and food safety [23]. Nevertheless, a substantial reduction in antibiotic usage is imperative for attaining a decline in AMR. For instance, the high level of antimicrobial resistance observed in *Escherichia coli* strains isolated from diarrhoeic piglets has been strongly associated with the routine use of postpartum antimicrobial therapy in sows [55]. This resistance is particularly notable in relation to commonly used treatments such as trimethoprim-sulphonamide. While the treatment of sick animals should not be delayed or avoided, the use of antibiotics for any other purpose is often inappropriate. Not all bacterial infections require antibiotic therapy. Broad spectrum antimicrobials are frequently used before or instead of obtaining a confirmed diagnosis and antimicrobial susceptibility testing. These antimicrobials are then administered to all individuals in the group, even those without any signs of infection, solely on the basis of economic considerations [56].

### 3.2. Antimicrobials for Metaphylactic and Prophylactic Use

The term ’disease prevention’ is employed to denote the administration of antibiotics for the purpose of prophylaxis or metaphylaxis [41]. Metaphylaxis is defined as the administration of antimicrobials to animals with clinical signs of disease. The primary objective of metaphylaxis is to mitigate the risk of the propagation of the disease from the clinically affected animals to the other animals in close proximity [57]. Prophylaxis, in contrast, is directed at healthy animals with the objective of averting the onset of an infectious disease in instances where the introduction of a pathogen is anticipated on the basis of history, clinical judgement, or epidemiological knowledge [58]. Appropriate prophylaxis should be prescribed by a veterinarian at a therapeutic dosage and for a limited time, with the aim of reducing morbidity and mortality in high-risk populations and the overall use of antimicrobials. This is particularly important when preventing disease onset, as it avoids the need for longer and more intensive therapeutic treatments [59].

### 3.3. Antimicrobials as Growth Promoters

Moreover, the discovery of the growth-promoting properties of antimicrobials in farm animals has led to the practice of feeding sub-therapeutic doses of antimicrobials for prolonged periods [20]. This has subsequently become a component of the production systems developed in industrialised animal husbandry in numerous countries. The impact of the low-dose administration of antimicrobials on the growth rate, feed conversion, litter size, and milk yield in dairy cows has been demonstrated to have a significant benefit to animal production [59]. Research has shown that low-dose antibiotics can change the microbiota without killing the beneficial bacteria needed for efficient nutrient absorption [42]. Studies have also shown that the growth-stimulating effects of different antibiotics vary. For instance, virginiamycin promotes growth, whereas tetracycline is less effective. This suggests that they interact differently with the microbiota. Virginiamycin can affect the diversity and composition of bacteria in the rumen of cattle [43]. It has also been observed that different animal species respond in different ways to antibiotic administration, which has an effect on nutrient uptake for body mass [60,61]. Additionally, it has been reported that long-term administration of sub-lethal doses of antibiotics can promote the growth of resistant microbes and the dissemination of antimicrobial genes [58]. These genes have been observed to be capable of acting against not only the antimicrobial agent used, but also other antimicrobials. Another significant concern regarding the utilisation of antimicrobials in farm animals pertains to the potential implications for human health. A considerable proportion of antimicrobials authorised for veterinary use are classified as antimicrobial classes that are also employed in human medicine [62]. Examples include glycopeptides, polypeptides, macrolides, tetracyclines, beta-lactams, sulfonamides, and aminoglycosides. Despite the paucity of studies that have directly addressed this issue, there is an evident consensus in the scientific literature that there exist pathways for AMR to disseminate in both directions, i.e., from humans to food-producing animals and vice versa, through the exchange of resistant bacteria [63,64]. The most commonly cited route of transmission is through the consumption of food contaminated with AMR bacteria. These bacteria may be commensal in animals but pathogens in humans, or vice versa, or may be commensal in both species but subsequently confer resistance to critical pathogens for both humans and animals [65]. The evidence base concerning the transmission of antibiotic resistance between humans and animals is limited and remains challenging to quantify, but concrete evidence exists in some cases. For instance, the rise in fluoroquinolone resistance observed in animal *Salmonella* isolates has been associated with a subsequent increase in human infections through the consumption of contaminated eggs. Furthermore, a robust correlation has been demonstrated between the utilisation of fluoroquinolones in food-producing animals and the emergence of the resistance to ciprofloxacin in both animal and human isolates [62]. In the context of *E. coli* infections, of particular significance is the rapid global emergence of ESBL-producing strains, which has been observed not only in human infections but also in bacteria isolated from food-producing animals, including cows and chickens [66]. The use of avoparcin (a glycopetide analogous to vancomycin) as an antimicrobial growth promoter in poultry has been associated with the emergence of vancomycin-resistant Enterococci [67]. The existence of this resistance has been documented in both food animals and in the human population. A comparable association has been documented between the European Union countries’ prohibition of avoparcin in 1997 and a consequent decrease in resistance. [68].

### 3.4. Coordinate Action Against AMR: Global Action Plan

In view of the possible relationship between the use of antibiotics in animal husbandry and its consequences for human health, the Global Action Plan (GAP) was initiated in 2015 as a joint undertaking between the World Health Organisation (WHO), the Food and Agriculture Organisation (FAO), and the Office International des Epizooties (OIE) [69]. It recognises the necessity to address antimicrobial resistance (AMR) via a “One Health” approach, which emphasises the interconnectedness of human, animal, plant, and environmental health, thereby promoting multisectoral collaboration among stakeholders.

In 2016, the Global Action Plan received renewed support when world leaders adopted a high-level political declaration on AMR during the seventy-first United Nations General Assembly, committing to implementing the plan at the global, regional, and national levels. The plan is based on key objectives to combat antimicrobial-resistant bacteria. This includes raising awareness, optimising applications, and reinforcing surveillance and research [70]. The principal objective of the Global Action Plan is to curtail antimicrobial resistance by diminishing its propagation, and, where feasible, rectifying prevailing trends. This will help preserve the effectiveness of antimicrobial treatments for both the prevention and treatment of infectious diseases, while also reducing their overall impact on human and animal health.

However, policies for antimicrobials in animal production vary between countries. Whilst the use of antimicrobials as growth promoters is prohibited in certain countries, including the European Union and the United Kingdom, they are utilised extensively in other regions [48]. The use of antibiotics in farm animals is a multifaceted issue that requires a balanced, responsible approach in order to preserve their effectiveness for humans and animals [71]. It is, therefore, essential that sustainable, responsible farming practices, such as the implementation of prevention measures (e.g., vaccines) and the improvement of farming conditions, are employed in order to reduce unnecessary antimicrobials.

## 4. How Do Antibiotics in Livestock Impact Antibiotic-Resistant Human Infections?

The use of antibiotics in intensive animal farming for food production first emerged in the 1940s in the USA [54]. A decade later, the marketing of antibiotics as an addition to animal feed, with the aim of promoting growth, became globalised. The first molecules to be used were streptomycins and penicillins. In further observations, this practice has been demonstrated to be associated with an increase in antimicrobial resistance in both pathogens (*Salmonella* spp. and *Campylobacter* spp.) and commensals (*E. coli* and *Enterococcus* spp.) involved in zoonotic infections in certain regions [72,73]. The WHO Foodborne Diseases Epidemiology Reference Group (WHO FERG) estimated that 600 million infections and 33 million disability-adjusted life years (DALYs) are caused by foodborne diseases each year, resulting in 420,000 deaths, including 125,000 children under five years of age [74]. These data provide a comprehensive understanding of the global challenges posed by food safety. At present, foodborne and/or animal-borne diseases are considered to be among the principal causes of morbidity and mortality on a global scale [75]. In Europe, the first signs of potential dangers to human health from the use of antibiotics in animal husbandry were observed in 1960, with particular concern expressed in the United Kingdom [76].

**Figure 1:**
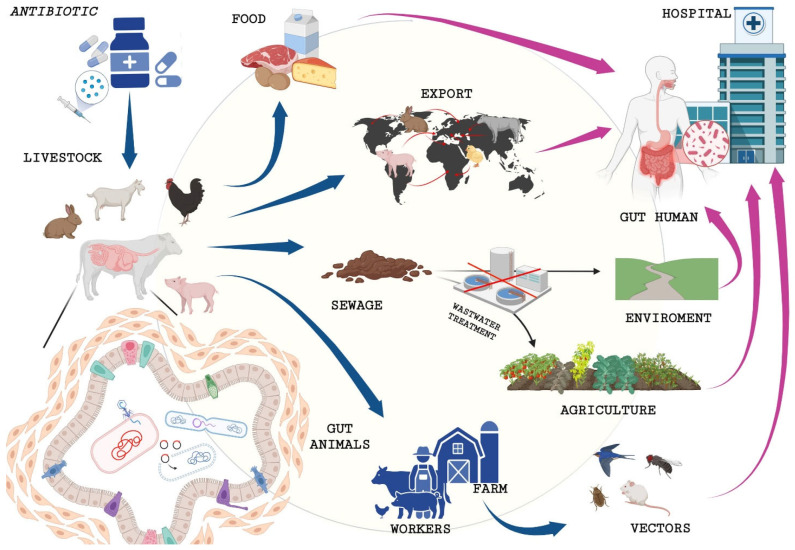
Pathways of antibiotic-resistant bacteria. Antibiotics used in veterinary medicine have the potential to contribute to the emergence of antimicrobial resistance (AMR) if not regulated effectively. The mechanisms by which resistance genes are transferred are more rapid in the gut tract of animals due to prolonged contact between different species, the greater number of microorganisms, and their close proximity. The following mechanisms have been identified: conjugation, transduction, and transformation. The subsequent transmission of these genes to bacteria can occur through diverse routes.

The initial evidence of the impact of antibiotic use in livestock farming was presented by the WHO in 1997, following an extensive data analysis [77]. The analysis yielded the following findings: (1) The use of antibiotics for growth promotion and animal health on livestock farms led to the emergence of bacteria resistant to multiple antibiotic classes, including those of clinical importance to humans. (2) These resistant bacteria and their associated genes can be transmitted to humans through direct contact or via derived foodstuffs [78,79] (Figure 1).

In 2005, the Food and Agriculture Organisation (FAO), the World Organisation for Animal Health (OIE), and the WHO established the importance of drawing up a list of Critically Important Antimicrobials (CIAs), which was subsequently renamed Medically Important Antimicrobials (MIAs) and subdivided according to their critical importance for humans [80]. This initiative was conceived as a mechanism to facilitate medical decision-making concerning antibiotic selection, thereby indirectly contributing to risk management and preserving the efficacy of these medications. The primary objective of this strategy was to contain the spread of MDR isolated from animals intended for food production [81]. In the MIA list, the various classes of antibiotics are subdivided into those exclusively designated for human use, those exclusively designated for animal use, and those designated for shared use between humans and animals [82]. A further subdivision, within the field of antibiotics whose use is shared between animals and humans, is characterised by three categories in descending order of importance: critically important, highly important, and important. It was decided that three antibiotic classes should be given top priority: fluoroquinolones, and third- and fourth-generation cephalosporins and polymyxins [82]. The emergence of the resistance to new antibiotics is unsurprising when considering that these medications are administered in farming contexts prior to their approval for human use, thereby compromising their efficacy. This phenomenon can be attributed to the complex regulatory framework that governs the evaluation of potential adverse effects associated with these novel pharmaceutical agents prior to their approval for human use [3,10]. Use in an agricultural or animal setting is a quicker method of recouping the costs incurred in the research, development, and production of the drug. A relevant example in the literature is the FDA’s approval of ceftiofur for animals before its approval for humans [52]. This led to a rapid acquisition of resistance to the cephalosporin class in human infections, which restricted its use. Antibiotic usage is a pivotal factor in this regard, with farming settings exhibiting higher usage than clinical settings. The duration of exposure also differs, with farming settings having a greater exposure time. This can be exacerbated by inadequate supervision by veterinarians or by the use of antibiotics that have been banned for this purpose [10,56,79].

## 5. Mechanisms of Antibiotic Resistance

### 5.1. Transmission Resistance

The main way AMR spreads is through the horizontal transmission of resistance genes, which involves the following: (a) conjugation (the transfer of DNA via mobile genetic elements); (b) transduction (bacteriophage-mediated gene transfer); and c) transformation (the retrieval of naked DNA from the environment and its incorporation into chromosomes via recombination) (Figure 1) [83].

The activation of these mechanisms enables microorganisms to respond rapidly to the pressure exerted by the suboptimal utilisation of antibiotics. Moreover, this results in the activation of internal microbiota-based signaling, leading to additional mutations that counterbalance the cost of maintained acquired resistance genes, while preserving their persistence (bet hedging) [8,83]. Gene transfer often involves replacing whole-gene cassettes with multiple antibiotic resistances, a process called “cross-resistance” [84]. This phenomenon is exemplified by methicillin-resistant *Staphylococcus aureus* (MRSA) strains, which carry the *mecA* or *mecC* gene (responsible for methicillin resistance) on a mobile genetic element called the staphylococcal cassette chromosome (SCCmec), which also harbours additional genes that confer resistance to other antibiotics [85]. This can result in a form of resistance selection, where exposure to the antibiotic in the cassette leads to resistance against various other antibiotics from different classes, which are then transferred together [84]. This contributes to the spread of MDR. *E. coli* (APEC) strains causing extraintestinal infections (e.g., pericarditis and sepsis) which have been isolated from broiler farm litter. These strains have been found to contain R-plasmids, IncC, and IncX1, which encompass genes that are resistant to a multitude of antibiotics [86]. In addition to these genes, the strains have been observed to harbour conjugal transfer system genes, insertion elements, and/or transposons. The following antibiotic resistance genes have been identified: for tetracyclines (*tet*(A)), aminoglycosides (*aph(6)*, *aph(3”)-Ib*, *aadA1*, aac(3), and *aph(3’)-Ia*), β-lactams (*CMY-2* and *TEM*), sulfonamides (*sul2* and *sul1*), and fluoroquinolones (*qnrS1*). These genes work with resistance mechanisms such as mutation, gene acquisition, efflux pumps, and DNA modification. The same plasmids have also been identified in other Gram-negative bacteria, including *Pseudomonas aeruginosa*, *Klebsiella pneumoniae*, and *Salmonella enterica*. Horizontal gene transfer (HGT) is a process that occurs most rapidly under conditions of high microbial density. This phenomenon is particularly evident in the case of the gut microbiota, both in the animal and human environments [87]. The use of sub-optimal amounts of antibiotics in livestock farming creates a selective pressure that favours the exchange of mobile elements between gut bacteria at a rate 25 times faster than that observed in the soil [88]. The microbiota is made up of many species of anaerobic bacteria that, despite their resistance, do not pose a threat. However, facultative anaerobic microorganisms in the *Proteobacteria* phylum could be pathogenic. Under selective pressure, these microorganisms may acquire new resistance genes from other bacteria in the microbiota [89].

### 5.2. Role of the Livestock Farms in the Spread of Resistances

Animals play a key role in the transmission chain, as shown by the presence of antibiotics and antibiotic-resistant bacteria in the environment, linked to faecal and urinary elimination [89]. A recent study has estimated that between 30% and 90% of the administered antibiotics are excreted with faeces, often in the form of unmetabolised compounds [90]. Intensive livestock farms are characterised by overcrowded conditions, frequently resulting in elevated animal population densities. In such environments, animals share space and resources, including water and food, and their waste, both solid and liquid, is accumulated in septic tanks or litter, in the case of poultry [77]. Several antibiotics have been found in poultry litter, including virginiamycin, salinomycin, penicillin, and bacitracin [91]. Residues of antibiotics are also found in waste, including residues from feed, food, and water used to clean the animals. This production process generates a large amount of antibiotic waste, which plays a significant role in the transfer of antibiotics to the environment [92,93]. The use of these materials as fertiliser is a well-established practice, but production sometimes exceeds demand, leading to the surplus being sent to disposal processes. This transfer of microbiological and genetic material can alter the existing microbiota. Studies have shown that diseases can be spread through food from crops infected with AMR microorganisms, due to manure from fertilisation or irrigation with surface water contaminated by runoff from animal waste [94]. In particular, manure has been shown to have a significantly negative impact on the environment (Figure 1). The issue is further exacerbated by the fact that less than 8% of the manure produced in Europe is treated, with significant regional variations being observed. In addition, there is a paucity of evidence that traditional treatments are efficacious in reducing the pathogen load [93]. Employing an array of analytical methods facilitated the differentiation of distinct transmission modes at each stage of the chain, from breeding through to sale. The transfer of bacteria carrying resistance genes from animals to humans can occur via several pathways [65]. Farm workers, who have direct contact with animals and their excreta, are at the forefront of direct transmission (Figure 1). Several studies have documented that these workers are the first to be exposed to AMR pathogens. Furthermore, in the absence of adequate personal hygiene and protective equipment, the clothing or surfaces worn or used by these workers may act as a vehicle for the spread of AMR bacteria within the home environment, with the risk of compromising the health of the resident community [11]. In conclusion, it is imperative that we emphasise that intensive livestock farming facilities are deficient in biosecurity measures. This allows insects and small animals to get in and out, including rodents and birds (Figure 1). These creatures can function as reservoirs and/or carriers for the transmission of AMR [95]. The transportation, slaughter, and consumption of animal products and their by-products (e.g., milk, eggs, and cheese) constitute additional pivotal phases in this sequence (Figure 1) [7]. Slaughter is carried out in facilities where a high volume of animals is slaughtered and processed on a daily basis. This process involves the movement of animals from one location to another while they are still alive, and even after they have been slaughtered, thereby facilitating the cross-transfer of resistant microbes [10]. Additionally, the waste produced during processing can be disposed of in canals, ultimately entering the environment (Figure 1). The dissemination of antimicrobial-resistant bacteria, particularly in under-resourced nations, has been attributed to the release of effluent from industrial facilities [19]. Whilst live microorganisms can be eradicated through standard wastewater treatment processes, the presence of DNA fragments, including AMR genes, remains a persistent concern [96,97]. Despite the development of innovative methodologies that address both pathogens and resistance genes, these approaches are not always integrated within conventional waste management practices [98]. The international trade of live animals has been identified as a potential contributing factor to the dissemination of antibiotic resistance genes. Exports of poultry, beef, and pig products have been identified as a significant source of these genes, with quantities so substantial that effective biological control becomes challenging without risking product spoilage [99]. This assertion is substantiated by a Danish study which revealed the prevalence of resistance to extended-spectrum cephalosporins in *E. coli* strains isolated from a chicken farm. The chickens in question had been imported from a farm in Scotland where the use of these molecules was permitted prior to 2012, and the resistance genes were thus introduced to Denmark via the chicken farm, despite the export ban [100].

The transmission of AMR bacteria from animals to humans has been well-documented, particularly in the case of infections caused by non-typhoid *Salmonella* spp., *Campylobacter* spp., and *S. aureus* [101]. A recent study has observed that 75% of human infections in recent years are of zoonotic origin [102]. Salmonellosis, a foodborne infection, has been observed worldwide, often resulting from the ingestion of infected meat (cattle, pigs, turkeys, and chicken), eggs, and milk [103]. The EFSA (European Food Safety Authority) and ECDC (European Centre for Disease Prevention and Control) have reported that, in 2021, out of a total of 2,201 cases, 590 (approximately 28%) were attributable to *Salmonella* spp. [101]. According to data from the United States-based FoodNet project (2022), the incidence of *Salmonella* and *Campylobacter* infections per 100,000 individuals was documented to be, respectively, 14.4 and 17.2. This incidence was predominantly observed among children under the age of five [104,105]. Furthermore, the EFSA has reported that a significant proportion of *Salmonella* spp. isolates from human infections in 2023 demonstrated resistance to ampicillin (21.3%), sulphonamides (20.8%), and tetracyclines (21.8%) [101]. Concurrently, *Salmonella* spp. strains were isolated from farm animals in a majority of European countries, exhibiting an equivalent percentage of resistance to the same antibiotic classes [101]. Furthermore, while a decrease in the percentage of resistance to ampicillin and tetracyclines was observed in human isolates during the period of 2014–2023, the percentage of resistance to ampicillin in isolates derived from broiler chickens showed an increasing trend throughout Europe [101]. Only 1.6% of *Salmonella* spp. were resistant to the third-generation cephalosporin cefotaxime, and only 1.3% to ceftazidime in human infections. These findings align with the low levels of resistance in animals: 0.2% in laying hens, 0.8% in pigs, and 1.4% in young cattle [71,101,106].

In 2022, a high prevalence of fluoroquinolone resistance was observed among *Salmonella* spp. isolated from broiler chickens (55.5% ciprofloxacin). In 2023, 21.8% of *Salmonella* strains isolated from humans demonstrated resistance to ciprofloxacin. From 2014 to 2023, there was a statistically significant increase in the resistance to ciprofloxacin in broiler and laying hens. Such increasing trends were also observed in *Salmonella* spp. isolates from humans in at least nine countries [101]. Minimal resistance to colistin has been recorded in *Salmonella* spp. isolates from farm animals and poultry carcasses [101]. According to EFSA data, in the 2022–2023 period, the resistance levels in *Campylobacter jejuni* isolates of human and animal origin to fluoroquinolones were sufficiently high to exclude this class of antibiotics from the recommended therapeutic treatment for humans [101]. An analysis of data from 2014 to 2023 revealed a significant increase in ciprofloxacin resistance among *C. jejuni* isolates from humans in 11 European countries. In 2022, 78.1% of *C. jejuni* isolates from turkeys and 70.9% from broilers exhibited resistance to ciprofloxacin [101]. The evaluation of methicillin resistance in *S. aureus* species, both in food and in animals, is inadequate due to its voluntary nature, and, consequently, not all countries have adopted it. The Livestock-Associated Methicillin Resistant *Staphylococcus aureus* (LA-MRSA) clone CC398, despite its low pathogenicity to humans, has the potential to serve as a reservoir for antimicrobial resistance genes that could be transmissible to virulent human *S. aureus* strains [107]. The levels of MRSA were observed to vary between species and countries, with isolation rates ranging from 0% in pigs in Norway to 80% in pigs in Belgium [101]. All isolates with methicillin resistance data from 2022–2023 carried the *mecA* gene [100]. But MRSA CC425 strains, carrying the *mecC* genes (primary host appears to be the European hedgehog), have been shown to have adapted to ruminants [108]. Germany and Denmark have reported an increased isolation of LA-MRSA strains that can cause human infections. Transmission through infected meat is a possibility [109,110]. However, CA and/or HA-MRSA lineages associated with human infections have been detected in farmed animals, suggesting an incidental exchange of strains between animals and humans [111]. There is little data on *E. faecalis* and *E. faecium*. The latter is more resistant to vancomycin than the former, but the difference is minimal in young cattle (age < 1 year) [101].

## 6. Policies and Strategies for Tackling Antimicrobial Resistance from Livestock to Humans

**Figure 2:**
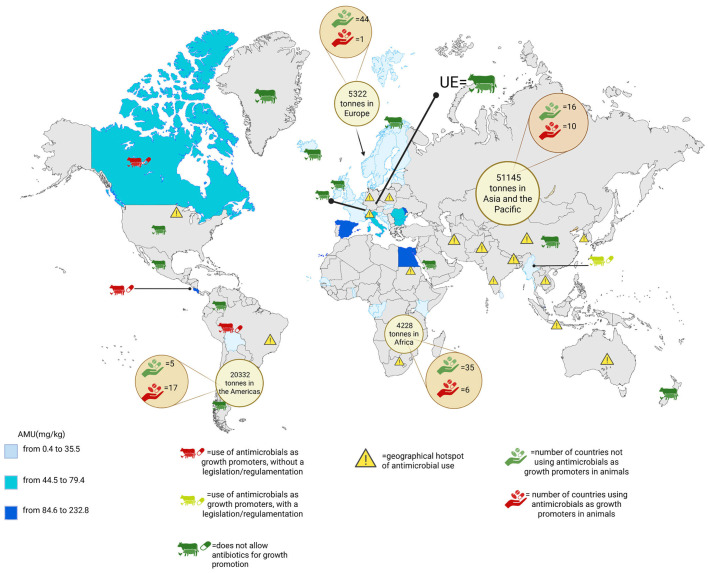
Global map integrating data on antimicrobial use (AMU) in food-producing animals. The most recent years of recorded AMU values were obtained from the WOAH AMU Data Portal. The 2022 data are available for the following countries: Finland, Ireland, Montenegro, the United Kingdom, and Senegal. The 2023 data set is from the following countries: Albania, Belgium, Bosnia and Herzegovina, Bulgaria, Croatia, Denmark, Estonia, France, Iceland, Italy, Latvia, Moldova, Norway, Romania, Slovakia, Slovenia, Spain, Sweden, Switzerland, the Netherlands, Myanmar, Sri Lanka, Bolivia, Canada, Costa Rica, Cuba, St. Vincent and the Grenadines, Cape Verde, the Republic of the Congo, Egypt, Gabon, Kenya, and Togo. Countries that use antimicrobials as growth promoters are highlighted, showing if they regulate this use (as in Myanmar—yellow icon) or not (as in Bolivia, Canada, and Costa Rica—red icon). Countries with legislation banning or restricting the use of antimicrobials as growth promoters are also indicated (e.g., Chile; China, Colombia; Greenland; Iceland; Saudi Arabia; the EU; the UK; and the USA).

As previously stated, AMR exerts a considerable influence on public health, primarily due to the transmission of antibiotic-resistant bacteria from livestock to humans and vice versa [10]. Consequently, numerous surveillance networks have been implemented in recent decades to systematically collect data on antibiotic usage and antibiotic resistance in livestock (Figure 2).

Antimicrobial quantities (in tonnes) intended for use in animals, as well as the number of countries reporting the use or non-use of growth promoters in 2022, were extracted from the WOAH Annual Report 2023 (https://amu.woah.org/amu-system-portal/amu-data, accessed on 19 April 2025). Furthermore, regions exhibiting elevated antimicrobial use intensity (i.e., ‘hotspots’ [48]) are delineated on the map. These regions include the following:

- Asia: eastern China, southern India, Indonesia, central Thailand, the eastern coastline of Vietnam, western South Korea, eastern India and Bangladesh, Pakistan, and north-west Iran;
- Europe: northern Italy, northern Germany, and central Poland;
- The Americas: south of Brazil and the Midwest of the USA;
- Africa: Nile delta and peri-urban areas of Johannesburg.

These networks are vital for government interventions as they allow the continuous monitoring of antibiotic usage, enabling the assessment of resistance trends across different animals [112]. The first country to establish a monitoring and research programme on antimicrobial resistance was Denmark (DANMAP) in 1995 [113]. The objective of DANMAP was to collect data on the sales of antibiotics for veterinary use. Reports from DANMAP highlighted a potential link between the widespread use of avoparcin in poultry and the contamination of meat with vancomycin-resistant enterococci. Subsequent to this, Denmark witnessed a surge in antibiotic usage, which exhibited a direct correlation with the emergence of *Escherichia coli* producing extended-spectrum β-lactamases (ESBL-producing *Escherichia coli*) isolates within the livestock population. Government interventions culminated in a decline in antibiotic consumption, concomitant with a concurrent decline in ESBL-producing *E. coli* cases in pigs. In 1996, US agencies initiated the monitoring of antimicrobial resistance in bacteria from humans, animals, and meat. The surveillance programme revealed the presence of fluoroquinolone resistance in *Campylobacter* samples from poultry. This finding was of such significance that it resulted in a reduction in the utilisation of enrofloxacin [114]. Furthermore, in 1997, the French National Observatory for Epidemiology of Antibiotic Resistance (RESAPATH) was established for the purpose of gathering data on antibiotic consumption in animals. The research revealed a 27.9% increase in antibiotic usage between 1999 and 2009, concomitant with a surge in cases of multi-resistant *E. coli* [115]. In 2002, Canada launched the Canadian Integrated Program for Antimicrobial Resistance Surveillance (CIPARS), a comprehensive surveillance system that integrates data on antibiotic use across both human and animal populations, with the objective of assessing its impact on public health. A notable finding from CIPARS was the identification of an increase in multidrug-resistant *Salmonella* in humans, which was found to be associated with the use of ceftiofur in poultry in specific regions of Canada [116]. This finding underscores the close correlation between antimicrobial resistance in humans and livestock, highlighting the need for interdisciplinary research and policy interventions to address these pressing public health concerns. China is a major producer and consumer of antibiotics, with an estimated production of 248,000 tonnes in 2013, 52% of which was allocated to livestock farming, approximately 2.8 times the amount used in the United States [117] (Figure 2). In 2019, China initiated measures to combat antimicrobial resistance by prohibiting the production, importation, and consumption of eight antibiotics. The following substances are proscribed: guaiacolin, methylene salicylic acid, bacitracin, bacitracin zinc, chlortetracycline, aflatoxin, virginia, and quinenone. These substances were previously employed as growth promoters and for the prevention of infection in livestock [118,119]. Japan was the first nation in Asia to establish a reporting system, the Japanese Veterinary Antimicrobial Monitoring System (JVARM), which commenced data collection in 2000 [120]. Since 2005, the European Surveillance of Veterinary Consumption (ESVAC) group of the European Medicines Agency has been responsible for the collection of data on the utilisation of veterinary antibiotics, with the initial findings published in 2011. This inaugural publication utilised sales data from eight countries and was expanded in 2017 to encompass all European countries [121] (Figure 2). These measures have included the prohibition of the use of certain antibiotics for non-therapeutic purposes and the development of stricter veterinary prescriptions to prevent overuse.

### Alternative Strategies to Tackle Antimicrobial Resistance

**Figure 3:**
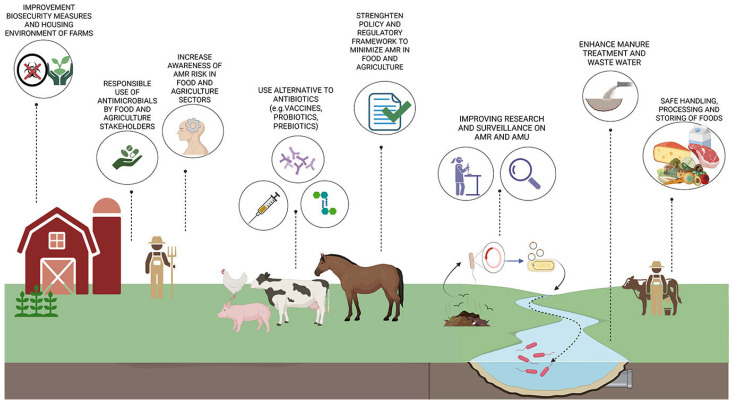
Strategies for combatting antimicrobial resistance.

Further investigation is necessary in order to determine the effectiveness of probiotics and prebiotics in reducing drug administration and to explore the potential application of veterinary vaccines. In the future, should technological advancements render this a possibility, genetic modifications could be used to develop species that are resistant to infection. The development of transgenic chickens that do not transmit avian influenza can be cited as an example of this phenomenon [122]. Alternative strategies (not antibiotics) are needed to promote animal health and prevent diseases (Figure 3).

A number of key actions must be taken in order to reduce the occurrence of antimicrobial resistance, and to curtail the emergence and subsequent propagation of resistance in food and agricultural sectors worldwide. The aim of these efforts must be to ensure the preservation of the efficacy of antimicrobials in the treatment of both animal and human infections.

Antimicrobials should be part of a comprehensive health management programme, not used separately. Vaccines are a promising alternative to antibiotics. They can reduce the need for antimicrobials, preventing bacterial disease or viral infections. They can also lower antibiotic use by reducing the risk of misdiagnosis and preventing secondary bacterial infections. However, many currently available veterinary vaccines have limitations, reducing their usefulness. For example, the vaccine may not match the circulating field strains for *Streptococcus suis*, swine influenza virus, *Haemophilus parasuis,* and *Eimeria* [123,124,125,126]. In other cases, protection after vaccination may be short-lived and require frequent booster vaccinations, as with *Clostridium perfringens* and bovine respiratory syncytial virus [127].

It has also been observed that different animal species respond in different ways to antibiotic administration, which has an effect on nutrient uptake for body mass [60,61]. It is crucial to note that the absence of illness is fundamental. To illustrate this point, consider a case of a banal intestinal infection in a farm chicken, which has a growth cycle of 40–45 days. In such a scenario, a loss of 1–2 days of nutritional intake due to diarrhoea can result in weight loss, which may have significant economic consequences. Many alternative substances are being investigated as replacements for antibiotics as in-feed growth promoters. Natural dietary supplements such as in-feed enzymes, probiotics, and prebiotics show promise (Figure 3). Enzymes help the animals break down and digest plant materials that they otherwise cannot utilise [128]. Broiler chickens have improved nutrient uptake, enhanced weight gain, and better feed conversion ratios when supplemented with enzymes. However, the response to food enzymes is influenced by factors like feed humidity, pH, and length of interaction [129]. A significant effect of probiotics is their capacity to compete with pathogens for adhesion sites along the intestinal mucosa, a phenomenon referred to as competitive exclusion. By establishing themselves in high numbers, probiotics limit the space available for harmful microorganisms, preventing colonisation and infection [130]. Probiotics boost the immune system, stimulating immune cell activity to improve surveillance and accelerate reactions against potential threats. Probiotics also influence the intestinal metabolic functions, promoting bioactive compounds that can combat pathogens [131]. A variety of microbial strains are employed in probiotic formulations. These include *Lactobacillus* and *Streptococcus* spp., which have been the focus of substantial research due to their recognised beneficial effects on gut health. Prebiotics, such as fructooligosaccharides (FOS), galactooligosaccharides (GOS), inulin, and mannan-oligosaccharides (MOS), are non-digestible fibres that stimulate the growth of beneficial microbes, such as *Bifidobacterium* and *Lactobacillus*, while preventing the presence of pathogens that cause gastrointestinal diseases, including *C. perfringens* [132]. Conversely, alternatives to antibiotics, such as antimicrobial peptides, phytochemicals, and organic acids, when incorporated into animal diets, have the potential to diminish the reliance on antimicrobials and, thereby, contribute to the reduction in antimicrobial resistance [133]. Unlike in a clinical setting where there are systems to control infections and health workers, there are no regulations about the risks of pathogens for workers living with animals. There is no collection of data on exposures and diseases to determine the risk and disease burden. Biosecurity and management practices are important in disease prevention, improving animal health and reducing the risk of pathogens [134]. The following elements are of particular significance in the context of biosecurity on farms and in livestock production:

- The implementation of controlled access measures for people, vehicles, and animals that may potentially carry pathogens;
- The maintenance of adequate farm fencing to prevent contact with stray and wild animals;
- The adherence to stringent hygiene standards, including regular handwashing, boot disinfection, and thorough cleaning of equipment, to minimise the spread of pathogens within farm environments. Effective sanitation protocols are essential for disinfecting surfaces, tools, and equipment to minimise the risk of disease transmission (Figure 3). The regular monitoring of animal health and behaviour allows for the early identification of potential illnesses, enabling timely interventions to prevent infection [135]. Maintaining good ventilation and not overcrowding animals are important strategies to improve air quality and support animal health. Regular monitoring allows farmers to quickly report any issues to the relevant authorities. This helps to contain outbreaks. Surveillance and research are vital for guiding stakeholders on how to slow the spread of antimicrobial resistance. They provide reliable data on antimicrobial-resistant microorganisms, antimicrobial use, and the presence of antimicrobial residues in food and feed [8]. Meat slaughter plants are another vulnerable sector. A study of a chicken processing plant revealed that biofilms can diminish the efficacy of cleaning procedures involving various chemicals. Furthermore, while urban plants are subject to regulatory standards for pathogens in wastewater, in certain countries, restrictions on the treatment of waste from production animal farms are either ineffective or have only recently been prohibited (i.e., in China). Whilst storage and composting are effective methods for separating solid waste from liquid waste, they do not reduce pathogens or pollutants. Calcium oxide stabilises solid waste before using it as fertiliser. The composition of wastewater renders conventional treatment methods ineffective, as evidenced by the frequent presence of elevated concentrations of antibiotics in post-treated European samples. Recent advancements in wastewater treatment technologies have been demonstrated by the adoption of adsorption methods utilising wheat straw, which have achieved a 90–98% removal of ciprofloxacin [136]. A range of alternative techniques is also being reviewed, including membrane separation, microbial electrolysis, photocatalytic degradation, and advanced oxidation. However, the implementation of these methods requires efficiency per cycle, safety during use, storage, and transportation, and the treatment of secondary polluting products, which would lead to increased costs [136]. In recent years, there has been an increasing focus among researchers on the development of new strategies for tackling antibiotic resistance in animal husbandry. The objectives of these strategies are twofold: firstly, to improve animal health, and, secondly, to reduce the reliance on conventional antibiotics. These strategies include the use of peptides and nanoparticles with antimicrobial activity [137]. Antimicrobial peptides (AMPs) are a class of small molecules composed of fewer than 100 amino acid residues, which are naturally produced by most living organisms. These peptides have demonstrated broad-spectrum antimicrobial activity against bacteria, fungi, and protozoa. The mechanisms through which they exert their effects are diverse, targeting both intracellular and extracellular components, thereby strengthening the host’s immune defences against pathogens [138]. Research has shown that AMPs are effective at preventing infections. They have also been found to enhance the effectiveness of antibiotics, reducing the development of drug resistance [139]. In some cases, dietary supplementation with AMPs has also been observed to promote animal growth. For example, microcin J25 (MccJ25), a peptide produced by *E. coli* and found in animal faeces, has been shown to positively influence animal growth [140]. Another innovative and promising technology under investigation involves the synthesis of nanoparticles (NPs) [141]. The synthesis of NPs can be achieved through a variety of methodologies, including physical, chemical, or green synthesis methods. The classification of these particles can be categorised into two broad categories: organic and inorganic. The organic category includes liposomes, polymeric, and lipid nanoparticles, while the inorganic group includes quantum dots and metal/metal oxide nanoparticles. These can be used as animal feed additives, as disinfectants, or as vaccine adjuvants to boost immunity. Moreover, they can be utilised as standalone antimicrobial agents [142]. Several studies have demonstrated that NPs improve antimicrobial activity against antibiotic-resistant bacteria and inhibit biofilm formation [143]. However, although various nanoparticles are currently being used as feed preservatives, growth stimulants, and antibiotic replacements, important issues such as bioaccumulation in the food chain remain to be addressed [144].

## 7. Conclusions and Future Directions

The extensive use of antibiotics in animal husbandry has been shown to contribute to the selection of bacterial populations that are increasingly resistant to the drugs used to treat animal and human diseases. Furthermore, it has been definitively established that there are multiple transmission routes through which these resistant bacteria can be transferred to humans. The issue is further compounded by the presence of antibiotic residues in foodstuffs [145]. It has been demonstrated that the regular ingestion of low-dose antibiotics in conjunction with foodstuffs engenders a selective pressure on intestinal bacteria, thereby promoting the proliferation of antibiotic-resistant bacteria. This genetic information is then transferred to other bacteria, including pathogenic ones. In an effort to contain the development of antibiotic resistance, the scientific community has reached a consensus that the use of antibiotics should be reserved exclusively for the treatment of diseases, following a rigorous diagnosis. Furthermore, the implementation of mass treatments for both metaphylactic and, most significantly, prophylactic purposes should be avoided or strictly limited. However, it has been observed that these pharmaceuticals are frequently administered to all animals, whether diluted in water or mixed in food, a practice that is likely to result in unintended consequences, including the increased prevalence of antibiotic resistance. The mass administration of antibiotics to all animals, even those only a few days old, in the absence of any evidence of specific pathologies, is driven by the objective of maintaining maximum animal survival. Unfortunately, even where there are regulations prohibiting this use of antibiotics, such as European legislation banning preventive mass treatments on farms, data show that the majority of antibiotics sold for use in livestock in several countries are still used for mass treatments [48,76]. Nevertheless, the issue poses a threat to global public health, as the proliferation of antibiotic-resistant bacteria transcends the geographical boundaries of nation states. It is, therefore, imperative that we acknowledge the global nature of this problem and devise equally global solutions. In order to comprehend the veracity of this assertion, one merely needs to consider the recent pandemic. Despite the temporal and geographical distance of the primary occurrence, owing to the phenomenon of globalisation, its consequences have, nevertheless, had a universal resonance. From this standpoint, the global transportation of animals and goods is widespread, and, consequently, a phenomenon (e.g., a resistant bacterium) originating in Asia or Oceania can reach Europe within a remarkably brief timeframe. Moreover, the paucity of data regarding veterinary antibiotics poses a significant challenge in the estimation of the amount of antibiotics utilised in the context of livestock farming. The absence of a standardised approach to the collection and sharing of data has the effect of making it difficult to apply these partial data findings to the global context. These limitations compromise the precision of estimates, hindering effective measures to address antimicrobial resistance. It is evident that a reduction in the use of antibiotics in food animals will not be achieved without a multifaceted approach. Despite the ongoing utilisation of antibiotics in numerous developing countries with the aim of enhancing productivity, there are positive indications that this practice is being phased out. For instance, all UN policies on sustainable development include the objective of reducing antibiotic use from a farm-to-fork perspective. Phytochemical substances and antimicrobial peptides have attracted a great deal of research interest as an alternative to antibiotics. But it is important to note that the effectiveness of these compounds has mainly been evaluated in experiments in labs and controlled conditions, rather than on actual intensive farming systems. In order to derive the maximum benefit from these substances, it is essential that intensive animal breeding farms be incorporated into the testing process. This will ensure that the practical applicability of the additives is clearly demonstrated. It is imperative that we acknowledge that the prospective unfavourable consequences of antibiotic reduction in animal husbandry can be mitigated. In fact, there exists a plethora of alternative measures capable of maintaining high productivity whilst simultaneously ensuring animal health. The primary strategy entails the formulation and execution of a comprehensive plan to enhance environmental and nutritional conditions, biosecurity measures, hygiene practices, and disease surveillance mechanisms within extensive livestock farms. The second strategy encompasses the advancement of novel technologies capable of facilitating the production of novel and more efficacious vaccines against both viral and bacterial diseases afflicting intensively farmed animals. The development of vaccines tailored to specific pathogens, with the aim of preventing disease outbreaks, is an effective strategy for reducing the reliance on antibiotic use. This approach has been demonstrated to engender two principal benefits: firstly, the reduction of healthcare costs and, secondly, the enhancement of public health outcomes.

## Figures and Tables

**Table 1:** Classes of antibiotics used in veterinary medicine.

Antimicrobial Agents	Species	Use	Mode of Action	Bacterial Resistance
**AMINOCOUMARIN** Novobiocin	Poultry [32,33,34], Caprine [35,36], Ovine [35,36]	Novobiocin is for treating mastitis in animals.	Novobiocin blocks the action of the bacterial DNA gyrase enzyme. Its action is bacteriostatic at lower concentrations and bactericidal at higher concentrations.	Resistance is linked to mutations in the *topoisomerase II* gene.
**AMINOCYCLITOL** Spectinomycin	Bovine [36], Poultry [32,33,34], Caprine [35,36], Ovine [35,36], Swine [35,37]	Used for respiratory infections in cattle and enteric infections in multiple species.	Inhibition of protein synthesis by binding to the 30S ribosomal subunit. Spectinomycin exerts bacteriostatic activity.	Resistance has been linked to aminoglycoside 3’-adenylate transferase.
**AMINOGLYCOSIDES** Dihydrostreptomycin	Bovine [35,36], Caprine [35,36], Ovine [35,36], Swine [35,37]	Aminoglycosides have a broad spectrum of applications, including the treatment of the following: Septicaemias; Digestive infections; Respiratory infections; Urinary diseases.	After internalisation, aminoglycosides bind to the 30S and 50S subunits, stopping protein synthesis. They are effective against rapidly multiplying organisms. At low concentrations, they exhibit bacteriostatic activity.	The resistance mechanism consists of an enzymatic modification of the antibiotic molecule, which may be either plasmid-encoded or chromosomally mediated, into three major classes: Acetyltransferases; Nucleotidyltransferases; Phosphotransferases.
Streptomycin	Bovine [35,36], Poultry [32,33,34], Caprine [35,36], Ovine [35,36], Swine [35,37]			
**AMINOGLYCOSIDES + 2** **DEOXYSTREPTAMINE** Apramycin	Bovine [35,36], Poultry [32,33,34], Ovine [35,36], Swine [35,37]			
Fortimycin Kanamycin	Bovine [35,36], Ovine [35,36], Swine [35,37]			
Framycetin	Bovine [35,36], Caprine [35,36], Ovine [35,36]			
Gentamicin	Bovine [35,36], Poultry [32,33,34] Caprine [35,36]			
Neomycin Paromomycin	Poultry [32,33,34] Bovine [35,36], Caprine [35,36], Ovine [35,36], Swine [35,37]			
**AMPHENICOLS** Florfenicol Thiamphenicol	Bovine [35,36], Caprine [35,36], Poultry [32,33,34], Ovine [35,36], Swine [35,37]	They are used in the treatment of respiratory infections in cattle, pigs, and poultry. The florfenicol used to treat pasteurellosis as well.	These antibiotics are bacteriostatic. They bind to the 50S ribosomal subunit, stopping protein production in bacteria. May be bactericidal against *S. pseudopneumoniae*	Resistance is mediated by an efflux pump system, due to a specific transporter (Flo-R). These genes are encoded on mobile genetic elements such as plasmids, transposons, or cassette genes.
**ANSAMYCIN—RIFAMYCINS** Rifaximin	Bovine [35], Caprine [35,36], Ovine [35,36], Swine [35,37]	This class of antibiotics is indicated for a limited number of cases (mastitis), and there are few alternatives.	Rifamycins inhibit the synthesis of RNA in microorganisms by binding to DNA-dependent RNA polymerase subunits. It is particularly efficacious in the treatment of intracellular microorganisms.	Resistance is due to a chromosomal mutation in the *rpoB* gene. This normally codes for the bacterium’s RNA polymerase.
**BICYCLOMYCIN** Bicozamycin	Bovine [36]	They are used in the treatment of digestive and respiratory diseases in cattle.	NA	NA
**CEPHALOSPORINS FIRST** **GENERATION** Cefacetrile Cefapyrin	Bovine [35,36,38]	Cephalosporins are employed in the treatment of the following conditions: Septicaemia; Respiratory infections; Mastitis.	These antibiotics disrupt the synthesis of bacterial cell wall formation by interacting with a group of proteins known as the penicillin-binding proteins (PBPs). Antibiotic activity of beta-lactams is confined to organisms in the log phase of growth or during active multiplication. The different generations of cephalosporins vary with respect to their antibacterial spectra, beta-lactamase sensitivities, and pharmacokinetics.	The mechanisms of antimicrobial resistance are multifactorial. These mechanisms may comprise: Modification of the drug target, via alterations to the PBPs; Downregulation of porins, with reduction in cell permeability; Increased expression of efflux pumps; Production of degrading enzymes (cephalosporinase).
Cefalexin	Bovine [35,36,38], Poultry [32,33,34], Caprine [35,36], Ovine [35,36], Swine [35,37]			
Cefalonium Cefazolin	Bovine [35,36,38], Caprine [35,36], Ovine [35,36]			
**CEPHALOSPORINS** **SECOND GENERATION** Cefuroxime	Bovine [35,36,38]			
**CEPHALOSPORINS THIRD** **GENERATION** Cefoperazone	Bovine [35,36,38], Caprine [35,36], Ovine [35,36]			
Ceftiofur	Bovine [35,36,38], Poultry [32,33,34], Caprine [35,36], Ovine [35,36], Swine [35,37]			
Ceftriaxone	Bovine [35,36,38], Ovine [35,36], Swine [35,37]			
**CEPHALOSPORINS FOURTH GENERATION** Cefquinome	Bovine [35,36,38], Caprine [35,36], Ovine [35,36], Swine [35,37]			
**FUSIDANE** Fusidic acid	Bovine [35]	Fusidic acid is employed in the treatment of ophthalmic diseases in cattle.	It interferes with the function of elongation factor and inhibits protein synthesis at the 50S subunit of the ribosome. The action of fusidic acid is bacteriostatic against Gram-positive organisms, and bactericidal against *Staphylococcus aureus.*	The resistance mechanism consists in the binding of the FusB protein to both EF-G, associated with a molecule of fusidic acid, and to the ribosome. This creates conformational changes with release of both EF-G and the antibiotic.
**IONOPHORES** Lasalocid	Bovine [35], Ovine [35,36], Poultry [32,33,34]	Ionophores are used in the context of controlling intestinal parasitic coccidiosis (*Eimeria* spp.).	They are lipid-soluble molecules that can bind and transport ions across cell membranes. They can change the concentration of different types of ions, including calcium, potassium, hydrogen, and sodium. This can block protein transport, reduce metabolic activity, and kill the organism.	Resistance to ionophores is probably adaptive, not due to mutation or gene acquisition.
Monensin	Bovine [35], Poultry [32,33,34], Caprine [35,36]			
Narasin	Bovine [35], Poultry [32,33,34]			
Salinomycin	Bovine [35], Poultry [32,33,34], Swine [35,37]			
**LINCOSAMIDES** Lincomycin	Swine [35,37], Poultry [32,33,34]	Lincosamides are used for treating the following conditions: Mycoplasmal pneumonia; Infectious arthritis; Haemorrhagic enteritis	Bind to the 50S subunit of bacterial ribosomes, suppressing protein synthesis via inhibition of peptidyl transferases. Present as bacteriostatic at low concentrations and bactericidal at high concentrations.	The resistance mechanism consists of methylation of the ribosomal subunit, by an enzyme of plasmid or chromosomal origin. Other mechanisms include increased activation of the efflux pump and destruction of the drug.
Pirlimycin	Swine [35,37]			
**MACROLIDES 14-** **MEMBERED RING** Erythromycin	Bovine [35,36], Poultry [32,33,34], Caprine [35,36], Ovine [35,36], Swine [35,37]	The following conditions are treated with macrolides: Mycoplasma infections in pigs and poultry; Haemorrhagic digestive disease in pigs; Liver abscesses and respiratory infections in cattle.	They inhibit protein synthesis by binding to the 50S ribosomal subunit. The action of these substances is bacteriostatic; however, at elevated concentrations, it can be bactericidal.	Resistance is observed at multiple levels: Alterations in the ribosomal structure present in Gram-positive bacteria; Increased expression of efflux pumps.
Oleandomycin	Bovine [35,36]			
**MACROLIDES 15-** **MEMBERED RING** Gamithromycin	Bovine [35,36]			
Tulathromycin	Bovine [35,36], Swine [35,37]			
**MACROLIDES 16-** **MEMBERED RING** Josamycin Terdecamycin	Swine [35,37]			
Kitasamycin Mirosamycin Tylvalosin	Poultry [32,33,34] Swine [35,37]			
Spiramycin Tilmicosin Tylosin	Bovine [35,36], Poultry [32,33,34], Caprine [35,36], Ovine [35,36], Swine [35,37]			
Tildipirosin	Bovine [35,36], Swine [35,37]			
**MACROLIDES C17** Sedecamycin	Swine [35,37]			
**ORTHOSOMYCINS** Avilamycin	Swine [35,37], Poultry [32,33,34]	Avilamycin is employed in the treatment of enteric diseases affecting poultry and swine.	It inhibits the proteins being built by blocking the A-tRNA site of ribosomal RNA 50S. It works against Gram-positive bacteria but not Gram-negative ones.	The molecular mechanisms underlying the resistance appear to be associated with mutations in single nucleotides at positions H89 and H91 of the ribosomal RNA 50S helices.
**NATURAL PENICILLINS** (including esters and salts) Benethamine penicillin Penethamate (hydroiodide)	Bovine [35,36]	This group of antibiotics has been proven effective in treating the following: Sepsis; Respiratory infections; Urinary tract infections.	These antibiotics disrupt the synthesis of bacterial cell wall formation by interacting with a group of proteins known as the penicillin-binding proteins (PBPs). This class of antibiotics is active against Gram-positive bacteria, and a limited number of Gram-negative bacteria.	Penicillins are susceptible to hydrolysis by beta-lactamases, also known as penicillinases. The combination of broad-spectrum penicillins and beta-lactamase inhibitors enhances the efficacy of treatment against both Gram-positive and Gram-negative pathogens.
Benzylpenicillin Benzylpenicillin procaine/ Benzathine penicillin	Bovine [35,36], Poultry [32,34], Caprine [35,36], Ovine [35,36], Swine [35,37]			
**AMDINOPENICILLINS** Mecillinam	Bovine [35,36], Swine [35,37]			
**AMINOPENICILLINS** AmoxicillinAmpicillin	Bovine [35,36], Poultry [32,34], Caprine [35,36], Ovine [35,36], Swine [35,37]			
Hetacillin	Bovine [35,36]			
**AMINOPENICILLIN +** **BETALACTAMASE** **INHIBITOR** Amoxicillin + Clavulanic	Bovine [35,36], Poultry [32,34], Caprine [35,36], Ovine [35,36], Swine [35,37]			
Acid Ampicillin + Sulbactam	Bovine [35,36], Swine [35,37]			
**UREIDOPENICILLIN** Aspoxicillin	Bovine [35,36], Swine [35,37]			
**PHENOXYPENICILLINS** Phenoxymethylpenicillin	Poultry [32,34], Swine [35,37]			
**ANTISTAPHYLOCOCCAL** **PENICILLINS** Cloxacillin Oxacillin	Bovine [35,36], Caprine [35,36], Ovine [35,36], Swine [35,37]			
Dicloxacillin	Bovine [35,36], Poultry [32,34], Caprine [35,36], Ovine [35,36], Swine [35,37]			
Nafcillin	Bovine [35,36], Caprine [35,36], Ovine [35,36]			
**PHOSPHONIC ACID** **DERIVATIVES** Fosfomycin	Bovine [35,36], Poultry [32,34], Swine [35,37]	The limited availability of this pharmaceutical product in numerous countries leads to its classification as a Very Hard to Import (VHIA) medication.	Fosfomycin functions as a competitive inhibitor of the substrate phosphoenolpyruvate (PEP) binding to the enzyme UDP-GlcNAc enolpyruvyl transferase (MurA). MurA catalyses the first committed step in bacterial peptidoglycan biosynthesis, which is thereby inhibited.	The following mechanisms are representative of resistance: Drug inactivating enzymes (FosA, FosB, FosC and FosX; of these, FosA has been found to be the most prevalent); Reduced absorption, associated with mutation in genes involved in the GlpT and UhpT transport systems.
**PLEUROMUTILINS** Tiamulin	Caprine [35,36], Poultry [32,34], Ovine [35,36], Swine [35,37]	They are used in respiratory infections in both pigs and poultry, as well as for the treatment of swine dysentery.	They act on the bacterial cell wall synthesis mechanism, inhibiting protein synthesis at the level of bacterial ribosomes. These antibiotics are active against Gram-positive bacteria, mycoplasmas, and anaerobes.	The development of resistance occurs through mutations to chromosomal targets. These occur in the 23S rRNA and *rplC* genes linked to bacterial ribosomes.
Valnemulin	Swine [35,37]			
**POLYPEPTIDES** Bacitracin	Bovine [35,36], Poultry [32,34], Swine [35,37], Ovine [35,36]	Bacitracin is employed in the treatment of necrotic enteritis in poultry. It is also indicated in the following: Septicaemia; Colibacillosis; Salmonellosis; Urinary infections.	The mechanism of action of this antibiotic involves the following: interference with cell membrane function; and suppression of peptidoglycan synthesis. Possess bactericidal activity.	Rare resistance cases reported.
Gramicidin	Poultry [32,34], Swine [35,37]			
**POLYMYXINS** Polymixin B	Bovine [35,36], Caprine [35,36], Ovine [35,36]	Polymyxin E (colistin) is employed in the treatment of Gram-negative enteric infections.	They are able to alter the phospholipid composition of bacterial cell membranes, compromising their permeability and functionality. It has been shown that polymyxins are more effective against Gram-negative bacteria than Gram-positive bacteria, with bactericidal action.	Polymyxin resistance is an exceptionally rare phenomenon, and is dependent on chromosomal mutations. However, a new plasmid-mediated gene, called *mcr-1*, has been discovered that confers resistance to colistin.
Polymixin E (colistin)	Bovine [35,36], Poultry [32,34], Caprine [35,36], Ovine [35,36], Swine [35,37]			
**QUINOLONES FIRST** **GENERATION** Flumequin	Bovine [35,36,38], Poultry [32,34], Caprine [35,36], Ovine [35,36], Swine [35,37]	They are used in the following conditions: Septicaemia; Colibacillosis.	Quinolones inhibit bacterial topoisomerase enzymes, in particular, the following: Topoisomerase II, also known as DNA gyrase; Topoisomerase IV. Their inhibition results in a reduction in DNA supercoiling and failure of DNA repair mechanisms. It has bactericidal activity.	Several resistance mechanisms have been detected: Chromosomal, with specific target mutations in the DNA gyrase and topoisomerase IV genes; Increased expression of efflux pumps and reduced expression of porins; Fluoroquinolone-resistant proteins (qnrA, qnrB, and qnrS) encoded by transmissible plasmids; AAC(6′)-Ib-cr, encoding an enzyme (acetylase); *qepA* gene, located on a plasmid, responsible for the production of an efflux pump capable of producing only hydrophilic fluoroquinolones.
Nalidixic acid	Bovine [35,36,38]			
Oxolinic acid	Bovine [35,36,38], Poultry [32,34], Swine [35,37], Ovine [35,36]			
**QUINOLONES SECOND** **GENERATION** **(FLUOROQUINOLONES)** Ciprofloxacin Difloxacin	Bovine [35,36,38], Poultry [32,34], Swine [35,37]	Fluoroquinolones are critically important in the treatment of the following: Septicaemias; Respiratory infection; Enteric diseases.		
Danofloxacin	Bovine [35,36,38], Caprine [35,36], Ovine [35,36], Swine [35,37]			
Enrofloxacin Norfloxacin	Bovine [35,36,38], Poultry [32,34], Caprine [35,36], Ovine [35,36], Swine [35,37]			
Marbofloxacin Orbifloxacin	Bovine [35,36,38], Swine [35,37]			
Ofloxacin	Poultry [32,34], Swine [35,37]			
**QUINOXALINES** Carbadox Olaquindox	Swine [35,37]	Quinoxalines (carbadox) is employed in the treatment of digestive diseases affecting swine, including swine dysentery.	NA	NA
**SULFONAMIDES** Phthalylsulfathiazole Sulfamonomethoxine Sulfamethoxine	Swine [35,37]	These classes alone or in combination are critically important in the treatment of a wide range of infections: Bacterial; Coccidial; Protozoal.	Sulfonamides are structurally similar to PABA (para-aminobenzoic acid). They competitively inhibit dihydropterate synthetase enzyme (DPS), interrupting the synthesis of dihydrofolic acid, precursor of the folic acid. They have bacteriostatic action, but can have bactericidal action at high concentrations. Diaminopyrimidines, such as trimethoprim, are able to inhibit dihydrofolate reductase. The combination with sulfonamide is synergistic, and enhances the antibiotic action.	Resistance mechanisms to sulfonamides can be divided into the following: Chromosomal resistance, attributable to mutations in the genes encoding dihydroptera synthase; Plasmid-mediated resistance, with the presence of mutations in dihydrofolate reductase.
Sulfacetamide	Bovine [35,36,39], Ovine [35,36]			
Sulfachlorpyridazine Sulfadimethoxazole	Bovine [35,36,39], Swine [35,37]			
Sulfadiazine Sulfadimethoxine	Bovine [35,36,39], Caprine [35,36], Ovine [35,36], Swine [35,37]			
Sulfadimidine Sulfamerazine	Bovine [35,36,39], Poultry [32,34], Caprine [35,36], Ovine [35,36], Swine [35,37]			
(Sulfamethazine, Sulfadimerazine) Sulfadoxine	Bovine [35,36,39], Ovine [35,36], Swine [35,37]			
Sulfafurazole	Bovine [35,36,39]			
Sulfaguanidine	Caprine [35,36], Ovine [35,36]			
Sulfanilamide Sulfaquinoxaline	Bovine [35,36,39], Caprine [35,36], Ovine [35,36]			
Sulfapyridine	Bovine [35,36,39], Poultry [32,34], Swine [35,37]			
**SULFONAMIDES+** **DIAMINOPYRIMIDINES** Sulfamethoxypyridazine	Bovine [35,36,39], Swine [35,37]			
Trimethoprim+ Sulfonamide	Bovine [35,36,39], Caprine [35,36], Ovine [35,36], Swine [35,37]			
**DIAMINOPYRIMIDINES** Baquiloprim	Bovine [35,36,39], Swine [35,37]			
Trimethoprim	Bovine [35,36,39], Caprine [35,36], Ovine [35,36], Swine [35,37]			
**STREPTOGRAMINS** Virginiamycin	Bovine [35,36], Poultry [32,34], Ovine [35,36], Swine [35,37]	It plays an important role in preventing necrotic enteritis (*Clostridium perfringens*).	These antibiotics bind to the 50S subunit of bacterial ribosomes to stop protein production by blocking peptidyl transferases.	Cross-resistance with macrolides and lincosamides is classified as macrolide–lincosamide–streptogramin B (MLSB) resistance due to their similar mechanisms.
**TETRACYCLINES** Chlortetracycline Doxycycline Oxytetracycline Tetracycline	Bovine [35,36], Poultry [35,36], Caprine [35,36], Ovine [35,36], Swine [35,37]	They are used in the treatment of chlamydial infections. Tetracyclines are of critical importance in the treatment of animals against heartwater and anaplasmosis.	The have the capacity to bind reversibly to the bacterial 30S ribosomal subunit, impeding the process of ribosomal translation at the aminoacyl-tRNA acceptor (A) site on the mRNA ribosomal complex. Possess bacteriostatic activity. They have bacteriostatic action, while, at high concentrations, they can have bactericidal capacity.	The phenomenon of resistance to antibiotics is associated with two distinct mechanisms. The first is characterised by the acquisition of efflux pumps, plasmid- or transposon-mediated. The second is attributed to the synthesis of a protective protein, which functions by either preventing binding at the ribosomal target.
**THIOSTREPTON** Nosiheptide	Swine [35,37]	This class is currently used in the treatment of some dermatological conditions.	Functions by impeding the formation of the 70S initiation complex. It has an inhibitory effect: - The initiation G protein IF2; - Elongation, by interfering with the G proteins EF-Tu, which is required for the rapid binding of the aminoacyl-tRNA to the ribosome; - EF-G, which catalyses the translocation of the tRNA-mRNA complex from the A and P sites to the P and E sites.	The mechanism of resistance consists of the target site modification, a process catalysed by an enzyme action of a methyltransferase (NHR) belonging to the class SpoU. In particular, we are witnessing 2’-O-methylation of the 23S rRNA, where the elongation (F-G) factor performs its function.

## Data Availability

Not applicable.
